# Alterations in the gut microbiota of patients with silica-induced pulmonary fibrosis

**DOI:** 10.1186/s12995-019-0225-1

**Published:** 2019-03-04

**Authors:** Yao Zhou, Lv Chen, Gaofeng Sun, Ying Li, Ruixue Huang

**Affiliations:** 1 Department of Occupational and Environmental Health, Xiangya School of Public Health, Changsha, 410078 China; 2 Department of Chronic and Non-communicable Diseases Control, City Center for Disease Control and Prevention, Urumqi, 830026 China; 3 Hunan Prevention and Treatment Center For Occupational Diseases, Changsha, China

**Keywords:** Silicosis, Gut microbiota

## Abstract

Silicosis resulting from silica exposure is a global occupational disease characterized by severe pathological changes in progressive pulmonary fibrosis. Previous evidence has indicated that dysbiosis of the gut microbiota occurs after environmental dust exposure and is associated with certain diseases. The aims of this study are to elucidate the compositional and functional characteristics of the gut microbiota in early-stage silicosis and to understand their influence on pulmonary fibrosis. We investigated the gut microbial composition of fecal samples from 18 patients and 21 healthy subjects using 16S rRNA gene sequencing technology. Compared with the healthy subjects, reductions in the levels of Firmicutes and Actinobacteria were noted in patients with silicosis and progressive pulmonary fibrosis, as well as lower levels of *Devosia,* Clostridiales*, Alloprevotella*and*Rikenellaceae_RC9. Lachnospiraceae* and *Lachnoclostridium* levels were increased in patients with silicosis. GOC and KEGG analyses were used to predict that certain bacteria taxa play critical roles in the development of pulmonary fibrosis, including posttranslational modification, amino acid transport and metabolism, nucleotide transport and metabolism, and ribosomal structure and biogenesis. KEGG analysis showed that certain taxa participate in various roles including cancer, endocrine metabolism, immune system, signaling molecules and interaction, and transcription. Collectively, in this pilot study, microbiota changes have been represented in the gut of patients with silicosis. Although this change in gut microbiota have been represented, caution is needed when interpreting the findings since this is observational finding, not necessarily causative. More studies should be performed in the expanding population to be verified and more studies underlying biological mechanisms for better understanding the relationship between gut microbiota and development of pulmonary fibrosis in patients with silicosis.

## Introduction

Silicosis, an occupational progressive fibrotic pulmonary disorder induced by inhaling free silicon dioxide or silica, is a public health concern that occurs commonly in many chemical and physical industries such as petroleum industries and iron and steel enterprises worldwide [1–3]. Silicosis is most common in low- and middle-income countries. In China, for instance, 600,000 cases of silicosis have been recorded over the past three decades, and this number continues to gradually rise each year, according to a report in 2016 [4]. In India, the rate of silicosis is estimated to range from 3.5 to 54.6% in various industries [5]. However, these values may be underestimated due to poor surveillance within developing countries. This public health concern is also prevalent in developed countries. The UK reported over 600,000 workers exposed to silica between 1990 to 1993, and silicosis-related deaths occur every year [6]. Workers in the US are also at a high risk of silica exposure, and silicosis-related deaths ranged from 3600 to 7300 annually between 1987 and 1996 [6]. Numerous studies have identified that inhalation of free silicon dioxide or silica is the cause of silicosis, and that the main clinical pathological feature is progressive pulmonary fibrosis [7]. The underlying mechanisms may involve silica-induced cytotoxicity of macrophages, activation of leukocytes to produce reactive oxygen free radicals, and damage to alveolar epithelial cells to induce fibroblast proliferation [8–10]. However, the exact mechanism of progressive pulmonary fibrosis in silicosis remains unclear.

Recent research on the lung–gut axis has revealed that the gut microbiota plays a critical role in maintaining pulmonary health, becoming a new frontier in the study of pulmonary-related diseases [11–13]. The gut microbiota, defined as the diverse microbial community that colonizes the host’s gastrointestinal (GI) tract [14], has been evaluated in various pulmonary disorders. Barcik et al. investigated the diversity of the gut microbiota in fecal samples obtained from healthy individuals and asthma patients; the results indicated that some bacterial strains are partially responsible for enhancing histamine secretion, with *Escherichia coli* and *Morganella morganii* significantly elevated in the gut microbiome of patients with asthma [15]. Dickson reported that “Even slight differences in the abundance of healthy gut bacteria have been implicated in diverse systemic diseases” [16]. For instance, butyrate, which is produced in the gut and provides primary energy for lung epithelial cells, which induce the development of regulatory T cells to dampen the immune response, is decreased in the guts of patients with respiratory disease [17]. The study by Tamburini and Clemente demonstrated that neonatal gut microbiota induced pulmonary immunity, helping prevent pneumonia, in neonates [18, 19]. Therefore, the gut microbiota has a role in maintaining lung health, and changes in gut microbiota diversity may lead to lung disease. Generally, many factors, including diet, lifestyle behaviors and environment, can modify the composition of the gut microbiota [20]. For example, alcohol is commonly considered to be a modulator of gut microbiota composition [21]. Dust in the home is an environmental factor that shapes the gut microbiota in infants infected with representative classes of bacteria(Actinobacteria, Bacilli, Clostridia and Gammaproteobacteria), showing a significant increase in dust–stool pairs compared with randomly permuted pairs [22]. Therefore, it was hypothesized that occupational exposure to silica modifies the composition of gut microbiota, which in turn is associated with progressive pulmonary fibrosis in patients with silicosis. To test this hypothesis, we performed microarray analysis of the fecal microbiota from patients with silicosis and from healthy individuals using 16S ribosomal RNA gene sequencing. Our results showed that patients with silicosis have a gut microbiota community profile distinct from that of healthy individuals. This knowledge may be useful for early diagnosis of silicosis and for developing a method to inhibit the pulmonary fibrosis.

## Material and methods

### Study design and recruitment of subjects

Eighteen male patients with silicosis were recruited from the Occupational Disease Prevention and Treatment Hospital from October 2017 to January 2018. The baseline general socioeconomic status was recorded including age, urban or agrarian resident, smoking status, ethnicity as shown in Table 1. Twenty-one sex-, age- and body mass index (BMI)-matched healthy individuals were also recruited as the control group. All patients with silicosis were diagnosed by at least three occupational disease physicians according to Chinese silicosis diagnostic standards (GBZ70–2015), based mainly on occupational exposure history, clinical symptoms, lung function investigation, X-ray examinations and silica levels reported in the workplace [23]. The inclusion criteria for the patients were males aged 18–70 years with symptoms of pathological fibrosis and their first diagnosis of silicosis without any history of clinical treatment as well as not use antibiotics and probiotics over the past sixmonths. The exclusion criteria for all enrolled participants were as follows: alcohol addiction; smoking history; complications of hypertension, diabetes, obesity, tuberculosis, asthma, chronic obstructive pulmonary disease or other related severe pulmonary diseases (e.g., lung cancer); BMI > 27 kg/m2; or use of antibiotics, probiotics, Chinese traditional herbal medicines or hormonal medications over the past 3 months. Twenty one healthy individuals underwent physical and liver biochemistry examinations, routine blood and urine tests, as well as serological tests to exclude any individuals with human immunodeficiency viral or hepatitis C or B viral infections and those with the abovementioned exclusion criteria.The silicosis process is divided into four phases clinically:Iphase,IIphase, IIIphase, and IVphase. The higher the phase, the more severity of silicosis is. Staging determination of silicosis requires assessment of the pneumonogram presentation including diffusionly distribute dots, small nodes and silicotic nodule. Considering silicosis is a development disorder and most of patients with IIIphase and IVphaseare treated with medication as well as accompanied with complications, we recruited Iphase and IIphase patients in this study.However, all silicosis patients, no matter how high the levels are, the presentation is lung fibrosis in common.

**Table 1:** Characteristics of enrolled subjects

Values		Patients with silicosis (A group)		Healthy controls (B group)		*P* value
		*N* = 18	%	*N* = 21	%	
Age(year)		56.7 ± 6.98		50.2 ± 8.32		0.35
Education level						
Elementary school(n)		3		4		
Junior high school(n)		15		14		
University or beyond(n)		0		3		0.43
Resident						
Urban		2		15		
Agrarian		16		6		0.06
Smoking						
Smoker		3		10		
Non-smoking		10		5		
Ex-smoker		5		6		0.17
Ethnicity						
Han nationality		16		18		
Minority		2		3		0.45
Marital status						
Married(n)		14		17		
Single/divorced/separated(n)		4		4		0.28
Occupation						
Machine operator(n)		5				
Mining operator(n)		11				
Metalworking(n)		2				
Administrator(n)				10		
Office worker(n)				11		0.04
BMI(kg/m^2^)		21.64 ± 7.5		22.7 ± 5.43		0.59
Silicosis phase	I	14	70			
	II	6	30			
Symptoms lasting time before diagnosis(Month)		11 ± 5				
Silica level	< 0.05 mg/m^3^	3	15			
	> 0.05 mg/m^3^	17	85			
Fung function	FVC(L)	2.44 ± 0.58		4.05 ± 0.89		0.04*
	FEV1(L)	2.45 ± 0.64		3.37 ± 1.01		0.04*
	MVV(L)	86.2 ± 6.47		121.3 ± 7.26		0.04*
X-ray result	Slight lung fibrosis	8	40			
	Severe lung fibrosis	12	60			
Platelet(10 × ^9^/L)		232.5 ± 51.9		234.6 ± 67.4		0.79
WBC(10 × ^9^/L)		5.53 ± 2.9		5.79 ± 3.04		0.58
Hb(g/L)		115.5 ± 49.4		139.2 ± 39.5		< 0.01

*WBC* white blood cell, *Hb* hemoglobin, *FVC* forced vital capacity, *FEV1* forced expiratory volume in the first second. MVV: maximueP value for age and BMI was evaluated by pearson chi-square test

* p<0.05

Above inclusion and exclusion criteria were created to make sure that the patients and control populations are similar in all terms except silica exposure. All enrolled subjects were informed of the nature of the study and were required to sign an informed consent form. All participants had the right to drop out of the study at any time without providing any explanation, and their information was kept confidential. The study has been approved by the Ethics Committee of Xiangya School of Public Health. The study was conducted adhered to standard biosecurity and institutional safety procedures.

### Fecal sample collection and DNA extraction

All participants in the study were required to undergo an overnight fast of at least 8 h in duration, after which blood (5 mL) samples were collected and stored at 4 °C for further routine blood and serological tests. All fecal samples were collected using disposable sterile forceps in the morning after at least an 8-h fast. If participants were unable to provide a fecal sample at the hospital, they had the option to collect the fecal sample at home and send it to the hospital on ice within 2 h. All fecal samples were immediately divided into aliquots and stored at − 80 °C for further DNA extraction.

Bacterial DNA extraction from the fecal samples was conducted at BioMARKER TECHNOLOGIES (Company, Beijing, China) using a Qiagen mini kit (Qiagen, Hilden, Germany). DNA was quantified using the Qubit 2.0 Fluorometer (Invitrogen, Carlsbad, CA, USA), and molecular size was estimated by agarose gel electrophoresis. All fecal microbial DNA was diluted to 10 ng/μL for microbial analysis.

The 16S rRNA gene was amplified by PCR using the following universal primers targeting the V3–V4 region of 16S rRNA: 338F 5′-ACTCCTACGGGAGGCAGCA-3′ and 806R 5′ -GGACTACHVGGGTWTCTAAT-3′. Each reaction contained 4 μL 5× Fast Pfu.

Buffer (TransGen Biotech, Beijing, China), 2 μL 2.5 mM dNTPs, 0.8 μL each primer (5 μM), 0.4 μL Fast Pfu Polymerase and 10 ng template DNA. Four PCR replicates were run per sample in a thermocycler (Eppendorf Mastercycler under the following parameters: 95 °C for 2 min, followed by 25 cycles of 95 °C for 30 s, 55 °C for 30 s and 72 °C for 45 s, and a final extension at 72 °C for 10 min. Reactions from the same sample were pooled, purified by agarose gel separation and band extraction (Axygen Biosciences, Union City, CA, USA), and quantified using a fluorometric kit (Quant-iTPicoGreen, Invitrogen).

### Species identification and classification

Bacteria16S:Silva (release 128, http://www.arb-silva.de) was used for species identification and analysis. Phylogenetic tree analysis was conducted using the neighbor-joining method, and multiple comparisons were performed using PyNAST (version 1.2.2, http://biocore.github.io/pynast/) and ClustalW2 [10] (http://www.ebi.ac.uk/Tools/msa/clustalw2/).

### Diversity analysis

#### Alpha diversity analysis

Alpha diversity is an index representing the diversity within a specific region or ecological system. Microbiota richness is commonly assessed using the Chao l and Ace estimators of species richness, and common measures of biodiversity are the Shannon–Wiener index and Simpson’s index. Higher diversity is represented by a higher Shannon–Wiener index and a lower Simpson’s index. The statistical analyses were conducted using Mothur software, version 1.30 (http://www.mothur.org/).

#### Beta diversity analysis

Beta diversity is an index used to analyze spatiotemporal variations in species composition. The species diversity was assessed using the binary Jaccard, Bray–Curtis and weighted UniFrac measures. Principal component analysis, principal coordinate analysis and relative analyses of environmental factors and samples (RDA/CCA) were performed using R software. Using principal component and principal coordinate analyses, the main parameters were identified and the differences among individuals or groups investigated. However, the former principal component analysis is the preferred method because it is based on the original species composition matrix, while the latter principal coordinate analysis is based on the distance matrix obtained from species composition arithmetic.

#### Cluster analysis

The unweighted pair group method with arithmetic mean was used for cluster analysis. The detailed procedure was as follows: the two smallest operational taxonomic units (OTUs) were clustered to form a new OTU, and then the mean distance between this new OTU and the other OTUs was calculated to find the next two smallest OTUs to cluster, continuing this process until all OTUs were clustered to form a phylogenetic tree.

#### Heat map analysis

The logarithm of each OTU was calculated, and the largest number of the top 80 was selected to perform heat map analysis using R software. In the heat map, each color lump represents a category richness of a fecal sample. The similarities in the microbiota community among fecal samples could be determined by the heat map.Mothur software (version 1.36.1)was used to obtain OTU table either in the study which was employed following the standard operating procedures from the website.

### LEfSe and Metastats analyses

LEfSe analysis (http://huttenhower.sph.harvard.edu/lefse/), a tool used to identify biomarkers, was used to estimate the effect of each species richness value on the variance. The aim of this method is to identify those species with the most significant variance in species richness between two groups. Metastats analysis (http://metastats.cbcb.umd.edu/) was performed to identify the potential species causing the variance between the two groups using t-tests, with *p*-values < 0.05, and Q-values< 0.05 were considered significant.

### Function and statistical analysis

COG (Clusters of Orthologous Groups) and KEGG(Kyoto Encyclopedia of Genes and Genomes) analyses were performed to predict the functions of the bacteria involved in the lung fibrosis in patients with silicosis compared with healthy subjects. COG database provide a tool to identification of orthologous genes which may be involved in various housekeeping functions especially those for transport and metabolism [24]. KEGG pathway analysis, is a predictive functional analysis using PICRUSt (phylogenetic investigation of communities by reconstruction of unobserved states) [25]. R software was used for all statistical analyses and construction of graphs. Values are presented as means and standard deviations, and t-tests were used to assess the significance of differences in microbial taxa, clinical parameters and diversity indices. *p* < 0.05 was considered to indicate significance.

## Results

### Characteristics of the study participants

In the study, 18 patients with silicosis and 20 healthy individuals were enrolled after applying the inclusion and exclusion criteria. No significant differences were found between the two groups in terms of age, BMI and platelet and white blood cell counts. However, hemoglobin levels were found to be significantly different between the two groups (*p* < 0.01) (Table 1).

### Gut microbial diversity in patients with silicosis compared with healthy subjects

**Fig. 1:**
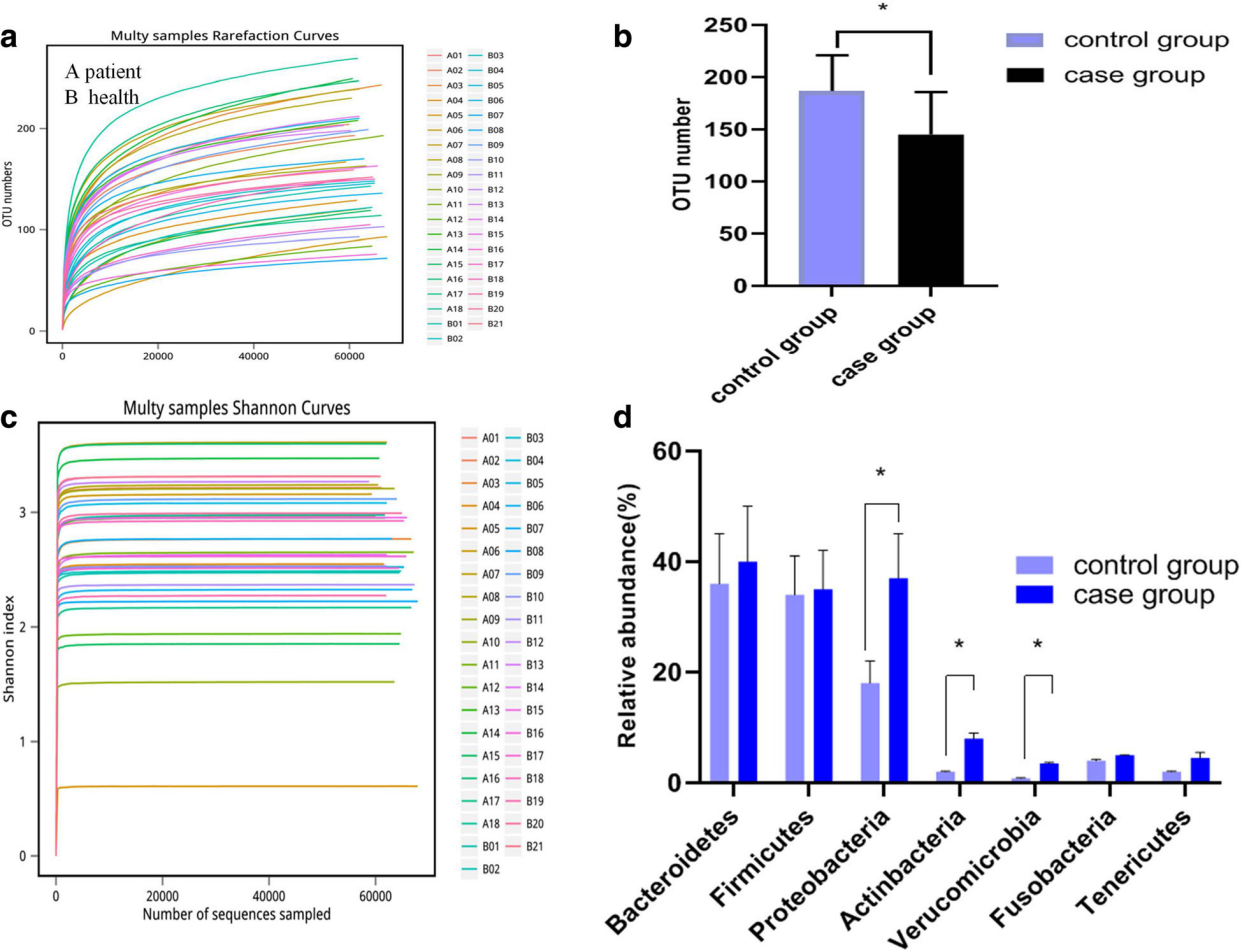
Alternations in the gut microbiota of patients with silicosis compared with healthy subjects. **a**, **c** Rarefaction curves comparing the species richness (observed OTUs) and overall diversity (Shannon–Wiener index). The curves indicated a lower diversityin the gut microbiota of patients with silicosis and lung fibrosis. **b** OUT number distribution in control and case group, respectively. **d** relative abundance(%) of bacteria in phylum level in two groups

To explore whether patients with silicosis exhibit differences in their gut microbiota compared with healthy subjects, the DNA from fecal samples was sequenced and assessed using IM_TORNEDO [26]. Figure 1a shows the number of OTUs detected in each sample from the patients and controls. The results suggested a different OUT number in patients with silicosis compared with healthy subjects (Fig. 1b). The Shannon–Wiener diversity index, used to evaluate species richness and evenness, was lower in the patients with silicosis compared with the control group (Fig. 1c), and Fig. 1d shows at phylum level, relative abundance(%) of proteobacteria, actinbacteria and verucomicrobia were increased in case group compared to control group.

**Fig. 2:**
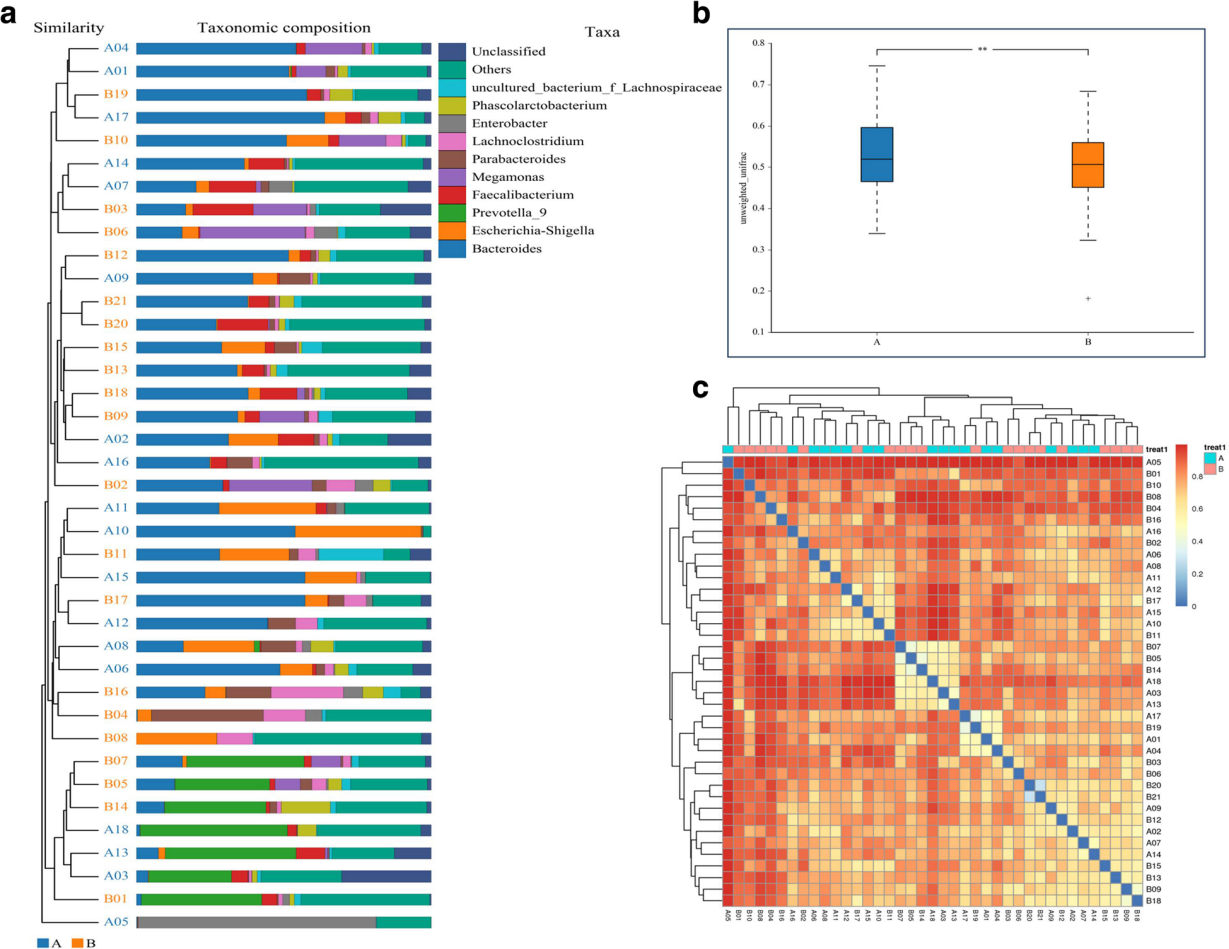
Beta diversity analysis of gut microbiota among patients with silicosis and healthy subjects. **a** A cluster tree showing significant gut microbiotadiversity in patients with silicosis. **b** Boxplots depicting differences in gut microbiota diversity between the two groups. Box parameters, the “-” symbol represents the median value, and the upper and lower ranges of the box represent the 75th and 25th percentiles, respectively. **c** A heat map depicting the relationship between the two groups. The blue color represents the range of 0–0.4 and the red color the range of 0.4–1.0

To explore spatiotemporal changes in species, beta diversity analysis, including cluster trees, distance boxplots, and heat map analysis, was performed based on the Bray–Curtis distance matrix. Significant differences in gut microbiota diversity were observed between healthy subjects and patients with silicosis (Fig. 2).

### The abundances of certain bacteria are associated with silicosis

**Fig. 3:**
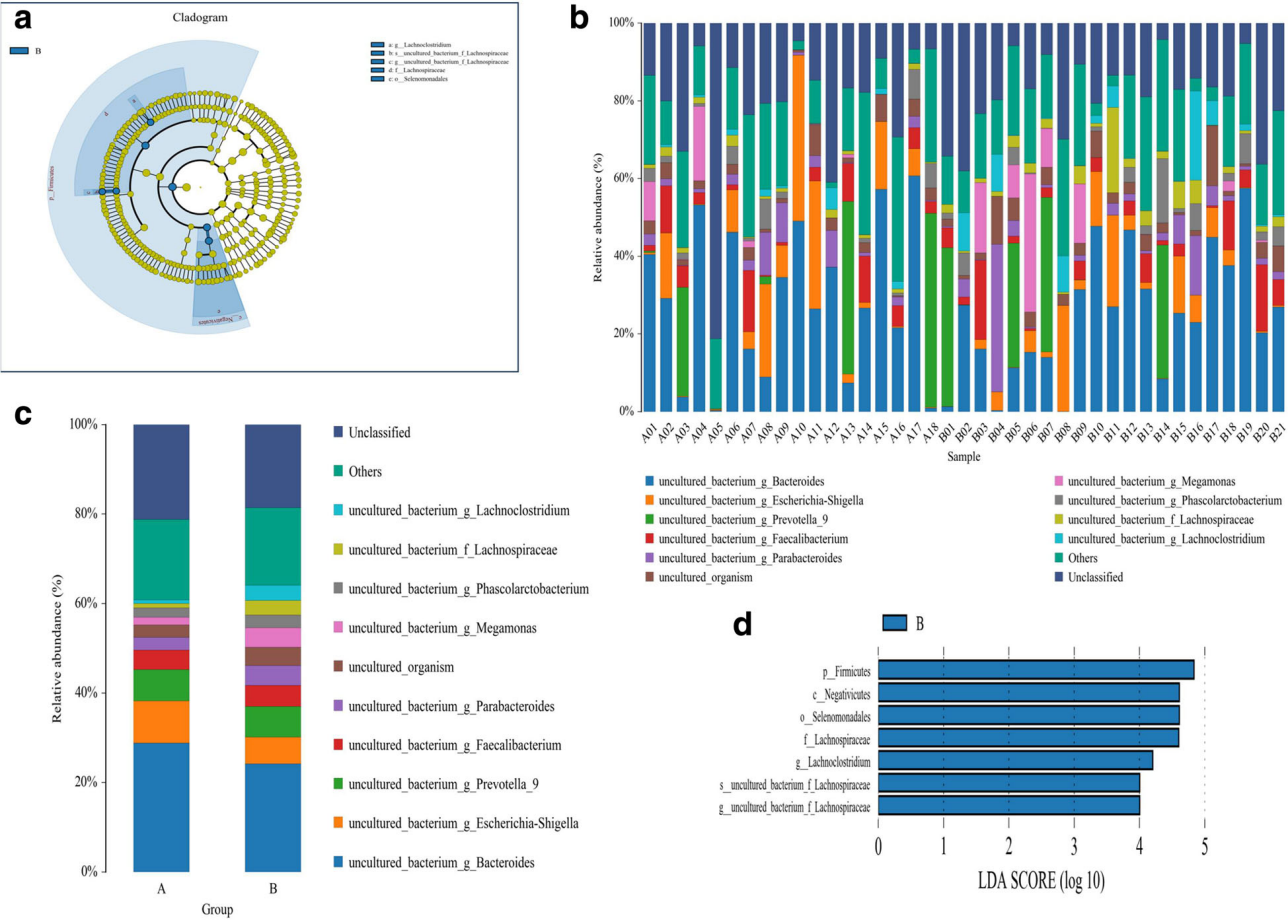
The abundances of certain bacteria are associated with silicosis. **a** A cladogram showing the gut microbiota community structures as well as the different predominant bacteria between the two groups. **b** Seven discriminating features obtained by LEfSe analysis indicating different bacterial abundances between the groups. **c** The abundances of the fecal species *Bacteroides* and *Escherichia-Shigella* were decreased in the guts of patients, whereas those of*Megamonas*,*Lachnospiraceae*,*Lachnoclostridium*,and *Parabacteroides* were increased at genuslevel (**c**–**d**). **d** The features indicating different abundances in the fecal samples between the patients with silicosis and healthy controls (LDA score > 4, p < 0.05, **d**)

To explore the specific bacterial taxa associated with silicosis, the LEfSe and Metastats methods were used to compare the fecal microbiota composition between the two groups. The cladogram in Fig. 3a shows the gut microbiota community structures and the differences in in the predominant bacterial taxa between the two groups. Briefly, the LEfSe analysis revealed seven discriminating features indicating different bacterial abundances in fecal samples between the patients and controls (LDA score > 4, *p* < 0.05, Fig. 3b).

**Fig. 4:**
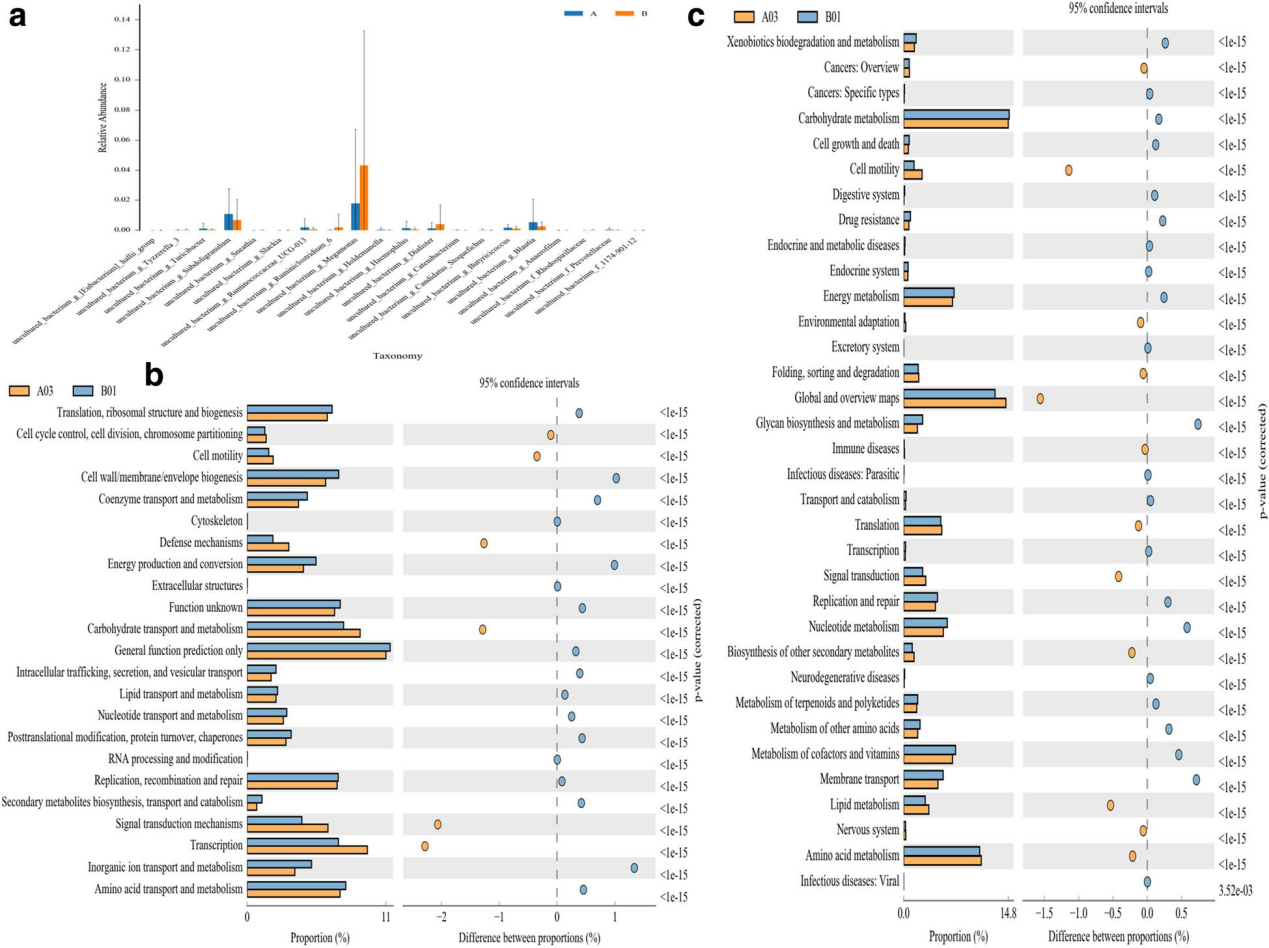
Function prediction. **a** Relative abundance analysis at species level in the fecal samples. **b** COG analysis showed that the bacteria represented in the guts of patients with silicosis were mainly involved in the following biological processes: cell wall/membrane/envelope biogenesis, carbohydrate transport and metabolism, transcription, inorganic ion transport and coenzyme transport and metabolism. A03 means the third sample in control group, B01 means the first sample in case group. **c** KEGG prediction analysis showed that the bacteria represented in the guts of patients with silicosis were mainly involved in the following biological processes: xenobiotic biodegradation and metabolism, cell motility, translation, nucleotide metabolism, metabolism of terpenoids and polyketides, and signal transduction and membrane transport. A03 means the third sample in control group, B01 means the first sample in case group

The fecal abundances of *Bacteroides* and *Escherichia-Shigella* were decreased in the gut of the patients, whereas those of *Megamonas, Lachnospiraceae, Lachnoclostridium,* and *Parabacteroides* were increased (Fig. 3c–d). These taxa may be potential patheogenesis for silicosis. Changes in the gut microbiota composition of patients with silicosis were also explored using the Metastats method. Table 2 shows the differential taxon abundances between patients and healthy subjects at the phylum level. At the phylum level, the abundancesof Actinobacteria, Acidobacteria, Gemmatimonadetes, Saccharibacteria, Fusobacteria, Aminicenantes and Verrucomicrobia were lower in patients with silicosis than in healthy subjects (p < 0.05), whereas those of Proteobacteria, Synergistetes, Lentisphaerae, Tenericutes and Cyanobacteria were higher in patients with silicosis than in healthy subjects (p < 0.05). At the species level, the abundances of *Subdoligranulum, Blautia, prokaryote, Nitrosomonadaceae, Bifidobacterium* and *Pseudomonas_aeruginosa* were decreased in the fecal samples of the patients, whereas those of *Megamonas,Dialiste* and *uncultured_bacterium_g_Ruminiclostridum_6* were significantly increased compared with the healthy controls (Fig. 4a). These data demonstrate different abundances of certain bacteria in the gut microbiota ofpatients with silicosis compared with healthy subjects, and the gut dysbiosis in patients with silicosis may be associated with the aberrant fecal microbiota composition.

**Table 2:** Differential taxa abundances between two groups at phylum level

	Healthy subjects(A)			Patients with silicosis(B)			P value	Q value
	Mean	Variance	SE	Mean	Variance	SE		
*Phylum*								
Firmicutes	0.329	0.0274	0.039	0.472	0.0170	0.0284	0.004	0.04
Actinobacteria	0.009	0.0004	0.004	0.001	0	0	0.0008	0.04
*Family*								
Veillonellaceae	0.0298	0.00248	0.0117	0.102	0.0168	0.0283	0.027	0.0753
Lachnospiraceae	0.136	0.014	0.0279	0.227	0.0172	0.0286	0.034	0.0887
Enterobacteriaceae	0.169	0.0447	0.0498	0.0777	0.00641	0.0175	0.0849	0.19
Enterococcaceae	0.0102	0.00171	0.0097	0.00005	0	0	0.169	0.297
Bacteroidaceae	0.324	0.0431	0.0489	0.272	0.0318	0.0389	0.42	0.559
Clostridiales_vadinBB60_group	0.0003	0.00000	0.0003	0	0	0	0.000999	0.00405
Leptotrichiaceae	0	0	0	0.00004	0	0	0.00099	0.00405
Succinivibrionaceae	0	0	0	0.00019	0	0.00019	0.00099	0.00405
Bradyrhizobiaceae	0.0002	0	0.0001	0	0	0	0.016	0.0518
Streptococcaceae	0.0043	0.00006	0.0019	0.00067	0	0.00022	0.02	0.0622
*genus*								
Devosia	0.0000147	0	0	0	0	0	0	0.00037
Ruminofilibacter	0.000014	0	0	0	0	0	0	0.00041
Bosea	0.000013	0	0	0	0	0	0	0.00044
Jatrophihabitans	0.0000131	0	0	0	0	0	0	0.00044
uncultured_bacterium_f_Blastocatellaceae_	0.0000145	0	0	0	0	0	0	0.00148
uncultured_bacterium_f_JTB255_marine_benthic_group	0	0	0	0	0	0	0	0.00216
Anaeroglobus	0.0000134	0	0	0	0	0	0.00013	0.00317

### Function prediction

COG and KEGG analyses were performed to predict the functions of the bacteria involved in the lung fibrosis in patients with silicosis compared with healthy subjects (Fig. 4b–c). According to the COG predictions, the bacteria represented in the patients with silicosis are mainly associated with the following biological processes: cell wall/membrane/envelope biogenesis, carbohydrate transport and metabolism, transcription, inorganic ion transport, and coenzyme transport and metabolism. According to the KEGG predictions, the bacteria represented in the patients with silicosis are mainly associated with the following biological processes: xenobiotic biodegradation and metabolism, cell motility, translation, nucleotide metabolism, metabolism of terpenoids and polyketides, signal transduction and membrane transport.

## Discussion

To the best of our knowledge, although the association between the gut microbiota and pulmonary disease has drawn much attention, and studies have been conducted on this association in certain lung diseases, including asthma, chronic obstructive pulmonary diseaseand viral lung infection [27, 28], the knowledge gap concerning the relationship between the gut microbiota and pulmonary fibrosis remains.

Emerging evidence has demonstrated that the human gut microbiota can confer either health benefits or a susceptibility to disorders.It has been reported that exposure to silica was associated with occurrence of systemic sclerosis [29]. Furthermore, Andreasson K et al. analyzed the prevalence of intestinal dysbiosis in systemic sclerosis patients and indicated that a majority(75.5%) of the patients exhibited intestinal dysbiosis, which was more severe in patients with pulmonary fibrosis [30]. Based on these previous studies, it is suggested that patients with silica-induced pulmonary fibrosis may exhibit dysbiosis of gut microbiota. Using 16S rRNA sequencing techniques, in this study, we have found possibilities that patients with silicosis have gut microbial changes. Our study revealed compositional differences in the gut microbiota of patients with silicosis compared with healthy subjects. At the phylum level,Firmicutes and Actinobacteriaabundances were lower in patients with silicosis (*p* < 0.05). The COG and KEGG prediction analyses showed the altered gut microbiota in patients with silicosis to be associated with numerous biological functions at the cell and systemic levels. Although this change in gut microbiota have been represented, caution is needed when interpreting the findings since this is observational finding, not necessarily causative.

The majority of the human gut/fecal microbiota is comprised of either the Firmicutes or Actinobacteria [31, 32]. Disruption of the balance between the Firmicutes and Actinobacteriamay increase the risk of many disorders, such as insulin resistance and obesity, as outlined in previous reports [33, 34]. In our study, we consistently observed a decreasing trend in levels of the Firmicutes and Actinobacteriain patients with silicosis with progressive pulmonary fibrosis. Although animal studies have linked gut dysbiosis with an increased risk of pulmonary fibrosis later in life [35], our study is the first to show a relationship between silica-induced progressive pulmonary fibrosis and altered gut microbiota diversity.

At the genus level, some studies have indicated that the species abundance level may be associated with maintaining body health. A decreased abundance of *Devosia*is associated with irritable bowel syndrome in humans [36]. Kelly et al. found an association between the risk of cardiovascular disease and *Alloprevotella* abundance, which was decreased in patients in the Bogalusa Heart study [37]. Our study is consistent with previous studies that showed lower levels of *Devosia, Clostridiales, Alloprevotella* and *Rikenellaceae_RC9* in fecal samples from patients with silicosis compared with healthy subjects. Decreased levels of these fecal bacteria may lead to a poor immune ability, resulting in failure to prevent progressive pulmonary fibrosis. However, it should be mentioned that in the LDA analysis in our study, no exciting and interesting differences showed between two group although the statistical results showed *p* value was under 0.05. This phenomenon might due to the small samples enrolled in this study, further investigations on larger participants’ samples should be considered to conduct the LDA analysis based on more larger population.

*Lachnospiraceae* and *Lachnoclostridium* levels were found to be increased in some diseases. A greater abundance of *Lachnospiraceae* was found in HIV-infected patients [38]. McCall et al. also found altered *Lachnospiraceae* levels in patients with Chagas disease [39]. *Lachnoclostridium* abundance was increased in patients who had been exposed to the antibiotic cefprozil [40]. Our study is consistent with these results, in that *Lachnospiraceae* and *Lachnoclostridium* levels were increased in patients with silicosis. The increased levels of *Lachnospiraceae* and *Lachnoclostridium* may contribute to gut disease susceptibility or inhibit metabolic pathways associated with progressive pulmonary fibrosis, eventually leading to silicosis. The detailed mechanisms by which the increased or decreased abundance levels of certain bacteria initiate the development of pulmonary fibrosis in patients with silicosis require further investigation.

A key advantage of this study is the prediction of bacterial functions using GOC and KEGG analyses. However, further mechanism studies should be performed to verify the roles of these potential bacteria in the development of pulmonary fibrosis.

Some limitations of the study should be mentioned. First, the samples sizes were small, which may affect explaining the conclusions. Large size of samples should be considered to further vilify the difference between case group and normal group. Second, some differences are relatively small rather than significant, this lead to the cautions while interpret these results. Third, dietary component for the patients which may have altered as a result of symptoms which in turn have altered the microbiota was not considered in this study. Forth, study in two groups according to our criteria would be possible affected by some cofounders, such as both two groups may be exposedto other pollutants such as particulate matter, location of housing, different childhood and companionships(e.g. originatingmore or less from agrarian culture), concomitant diseases and medications. For instance, ambient PM_2.5_ causes gut dysbiosis and may subsequently contribute to developing glucose metabolism abnormalities [41]. Geographical location, such as urban and rural areas, is associated with alterations of gut microbiota [42]. Therefore, for further study, we should formulate more critical inclusion and exclusion criteria to make the interpretation of results more reliable. In conclusion, we report biological community composition occurred differently in control group and silicosis patients.Moreover, the levels of Firmicutes and Actinobacteria were reduced in patients with silicosis. This may give us a deeper understanding on the possible role of gut microbiota in the pathogenesis of silica-induced pulmonary fibrosis.
